# InsulinAPP application protocol for the inpatient management of type 2 diabetes on a hospitalist-managed ward: a retrospective study

**DOI:** 10.20945/2359-3997000000496

**Published:** 2022-06-27

**Authors:** Marcos Tadashi Kakitani Toyoshima, Pedro Henrique Ribeiro Brandes, Gerhard da Paz Lauterbach, Jéssica Ribeiro Andrade Moraes, Edison Ferreira de Paiva, Guillermo E. Umpierrez, Marcia Nery, Rodrigo Hidd Kondo

**Affiliations:** 1 Universidade de São Paulo Faculdade de Medicina Hospital das Clínicas São Paulo SP Brazil Serviço de Onco-endocrinologia, Instituto do Câncer do Estado de São Paulo Octávio Frias de Oliveira, Hospital das Clínicas, Faculdade de Medicina, Universidade de São Paulo, São Paulo, SP, Brasil; 2 Universidade de São Paulo Faculdade de Medicina Hospital das Clínicas São Paulo SP Brazil Serviço de Endocrinologia e Metabologia, Hospital das Clínicas, Faculdade de Medicina, Universidade de São Paulo, São Paulo, SP, Brasil; 3 Universidade de São Paulo Faculdade de Medicina Hospital das Clínicas São Paulo SP Brazil Serviço de Medicina Hospitalar, Hospital das Clínicas, Faculdade de Medicina, Universidade de São Paulo, São Paulo, SP, Brasil; 4 Emory University Division of Endocrinology Department of Medicine Atlanta GA USA Department of Medicine, Division of Endocrinology at Emory University, Atlanta, GA, USA

**Keywords:** Type 2 diabetes mellitus, inpatients, hyperglycemia, insulin, hospitalists

## Abstract

**Objective:**

We assessed metrics related to inpatient glycemic control using InsulinAPP, an application available for free in Brazil, on the hospitalist-managed ward of our hospital.

**Subjects and methods:**

We performed a retrospective study of patients with type 2 diabetes (T2D) admitted from November 2018 to October 2019. InsulinAPP recommends NPH and regular insulins three times a day, in bolus-correction or basal-bolus schemes. Parameters that included BG within range of 70-180 mg/dL, insulin treatment regimen and frequency of hypoglycemia were evaluated.

**Results:**

A total of 147 T2D individuals (23% medicine and 77% surgery) were included (mean age 62.3 ± 12.7 years, HbA1c: 8.3 ± 3.0%). The initial insulin regimen was 50% bolus-correction, 47% basal-bolus and 3% with sliding scale insulin. During hospitalization, 71% patients required a bolus-basal regimen. In the first 10 days of the protocol, 71% BG measurements were between 70-180 mg/dL and 26% patients experienced one or more episodes of hypoglycemia < 70 mg/dL, and 5% with BG < 54 mg/dL.

**Conclusion:**

The results of this retrospective study indicate the InsulinAPP application using human insulin formulations was effective and safe for the management of hyperglycemia on a hospitalist-managed ward, with more than 70% BG measurements within the therapeutic range and a low rate of hypoglycemia.

## INTRODUCTION

Inpatient hyperglycemia is defined as blood glucose (BG) greater than 140 mg/dL ( 1 , 2 ). Observational studies indicate that in-hospital hyperglycemia affects 32% to 38% of non-critical patients ( 1 , 3 ). Early diagnosis and adequate treatment of hyperglycemia have been shown to reduce morbidity, length of stay (LOS), readmission rate, need for admission to intensive care unit (ICU), hospital costs, and hospital mortality ( 4 - 7 ). Despite this evidence, inpatient glycemic control remains unsatisfactory, particularly because of treatment complexity and fear of hypoglycemia ( 1 , 8 - 10 ).

Improvement in the quality of healthcare services is a major concern among hospital administrators. The World Health Organization recommends high-quality hospital services must be safe, effective and patient-centered ( 11 ). Management of hyperglycemia has received special attention as a metric of service quality ( 10 ), and hospitals are increasingly required to have protocols for inpatient management of hyperglycemia. However, a multicenter Brazilian study showed lack of standard protocols in most hospitals, and less than a third of patients with diabetes received adequate insulin therapy ( 4 ). Metrics related to glycemic control (glucometrics) can provide useful information to improve the quality of patient care ( 10 , 12 ). Common metrics used to assess the effectiveness of inpatient glycemic control include time in glycemic range, glycemic variability, and rate of hypoglycemia ( 13 ).

Diabetes technology tools including mobile communication technology and computerized insulin administration devices have shown to be promising in improving glycemic control and adherence to insulin therapy ( 14 ). Some computerized systems are commercially available to adjust insulin therapy in non-critically ill patients resulting in increased adherence to protocols and reducing hypoglycemic events ( 15 ). The InsulinAPP is a publicly available and free application to guide the initial insulin therapy orders and daily adjustment of human (NPH and regular) insulins for the management of type 2 diabetes in the hospital setting ( 16 ). This protocol was approved by hospital administrators as the standard of care for managing inpatient hyperglycemia.

The aim of the study was to assess metrics related to inpatient glycemic control using InsulinAPP protocol on the hospitalist-managed ward of *Hospital das Clínicas da Faculdade de Medicina da Universidade de São Paulo* , the largest public hospital complex in Latin America ( 17 ).

## SUBJECTS AND METHODS

### Study design

This retrospective study was carried out by reviewing the electronic medical record of patients with diabetes admitted to the hospitalist-managed ward of our hospital from November 2018 to October 2019. The study protocol was approved by the local ethics review committee (CAAE 17904819.2.0000.0068).

### Patients

The study included individuals with type 2 diabetes (T2D) treated with the InsulinAPP protocol for at least 48 hours. Patients with a known history of T2D or with glycated hemoglobin (HbA1c) greater than 6.5% on admission were included ( 2 ). We excluded individuals with type 1 diabetes, pregnancy, age < 18 years, end-of-life or palliative care, and participation in the protocol < 48 hours. A sequential and convenience sample was chosen, according to the inclusion and exclusion criteria. The reporting of this study conforms to STROBE guidelines ( 18 ).

### Characteristics of the hospitalist-managed ward

The hospitalist-led inpatient diabetes management program is an ideal model in institutional settings with limited endocrinology resources and champions the culture of inpatient glycemic management ( 19 ). Our hospitalist-managed ward is a highly dependency unit that was established in March 2018, an innovative project in our hospital, in which the hospitalist team became the main care team for patients with multiple comorbidities and high complexity. The hospitalist-managed ward has a total annual occupancy capacity of 9490 bed-days and an available computerized medical order entry (CPOE) system.

## TEAM EDUCATION

Before the institution of InsulinAPP protocol, in-person lectures and grand round presentations were held to nurses, internists, hospitalists, pharmacists, dietitians, medical students, residents and fellows to improve the team’s confidence in diabetes control and encourage adherence to the previous established protocol ( 16 ).

## DATA ACQUISITION

Clinical and laboratory data of patients were collected from electronic medical records. Patient follow-up data were collected: transfers to another ward or to the ICU, indication of palliative care, deaths and hospital discharge, including those transferred to another ward. The glomerular filtration rate was calculated using the Chronic Kidney Disease Epidemiology Collaboration equation (CKD-EPI).

### Standardized glycemic management

Point-of-care BG before the three main meals of the day (breakfast, lunch and dinner) were assessed until the tenth day of hospitalization. If the patient was fasting, the BG measurement routine schedule was held at least three times a day. BG concentrations between 70-180 mg/dL were considered within the therapeutic range. Hypoglycemia was defined as a BG less than 70 mg/dL, but BG <54 mg/dL and <40 mg/dL were also included in the analysis. HbA1c was measured in all patients on admission, unless the patient had the measurement within the last three months. NPH insulin was used three times a day (before breakfast, before lunch and at bedtime) as basal insulin and regular insulin was used three times a day (before meals) as prandial and correctional insulin. The inpatient glycemic management was performed according to protocol using InsulinAPP application ( http://www.insulinapp.com.br ). The InsulinAPP protocol was more detailed in the previously published article ( 16 ) and summarized in the Supplementary Material. If the T2D patient was using a total daily dose (TDD) of insulin greater than 0.2 U/kg/day or the initial BG greater than 250 mg/dL, the initial regimen of insulin therapy was the basal-bolus. Otherwise, the initial insulin therapy regimen was bolus-correction. If the individual is unaware of the diagnosis of diabetes on admission, but the HbA1c is above 6.5%, the data were included in the study. These patients without known diabetes and with hyperglycemia < 250 mg/dL who started the InsulinAPP protocol, the application kept the patient under surveillance for the first 24 to 48 hours and correction insulin regimen with regular insulin was performed when hyperglycemic episode had occurred (sliding scale insulin or SSI). If the patient started fasting, the dose of prandial insulin was interrupted. Thus, the basal-bolus regimen was changed to the basal-plus regimen (basal insulin plus correction) and the bolus-correction regimen was changed to SSI, while fasting was maintained. Patients with bolus-correction regimen and who maintained hyperglycemia during follow-up started to receive basal-bolus regimen.

## STUDY POPULATION

The study was carried out with a convenience sample size in which data collection was limited to one year of hospitalization.

### Outcomes measures

A descriptive analysis of data on glycemic control parameters, insulin treatment regimen, frequency of hypoglycemia and severe hypoglycemia.

### Statistical analysis

Data were shown in absolute and relative frequency, mean ± standard deviation (SD) or median (interquartile range). For relative frequency of outcome measures, we use as denominators the number of patients, the number of BG measurements, or monitored patient-day ( 20 ). Pearson’s chi-square test or Fisher’s exact test were used for categorical variables, and Wilcoxon rank-sum test or Kruskal-Wallis rank-sum test or repeated-measures ANOVA for continuous variables. Individual pre-prandial BG means were calculated from day 1 to 10 of the protocol. Repeated-measures ANOVA was used to compare pre-prandial BG means among different meals, and pairwise comparisons were performed with paired t tests, using Bonferroni correction for multiple analysis. No imputation method was used to address missing data. R software version 4.1.1 for Windows (R Project for Statistical Computing) was used for analysis. A *P* value <0.05 was considered significant.

## RESULTS

### Profile of the hospitalist-managed ward

From November 2018 to October 2019, the ward occupancy rate was 90%, totaling 8,156 bed-days and 7,353 patient-days. During this period, there were 571 total admissions, 398 discharges, 51 deaths and 679 transfers to another facility of the hospital complex. Patients with diabetes occupied 16% patients-day. Only patients with known diabetes or with signs and symptoms of hyperglycemia started the InsulinAPP protocol. The patients in the present study occupied 3043 beds-days, representing 37% of the total number of beds-days.

### Baseline characteristics

The InsulinAPP protocol was started in 174 patients. However, 19 individuals with stress hyperglycemia without diabetes, five admitted for exclusive palliative care, two did not complete the 48-hour protocol and one with type 1 diabetes were not included in the analysis. Out of 147 T2D (23% medicine and 77% surgery) patients included in the study, 95% patients had a known diagnosis of diabetes and 5% were newly diagnosed diabetes (HbA1c ≥6.5%). Clinical and laboratory data of individuals are shown on Table 1 . The most common admitting diagnoses in medicine patients were cardiovascular (32%), infectious (24%), neurological, gastroenterological and pulmonary (9%) disorders, while the most common types of surgery were vascular (83%), abdominal (9%) and neurosurgical (5%) procedures.

**Table 1:** Clinical and laboratory characteristics of individuals with type 2 diabetes, initial parameters of InsulinAPP protocol and follow-up

Variable			Type 2 DM (n = 147)
Sex			
	Male		87 (59.6%)
	Female		59 (40.4%)
Age, years			62.3 ± 12.7
BMI, kg/m^2^			27.2 ± 5.9
Admission cause			
	Medicine		34 (23.1%)
	Surgery		113 (76.9%)
eGFR, mL/min/1.73 m^2^			71.6 ± 30.7
Serum creatinine, mg/dL			1.3 ± 1.0
Admission BG, mg/dL			220.3 ± 92.9
HbA1c, %			8.3 ± 3.0
InsulinAPP protocol			
	Initial insulin regimen		
		Basal-bolus	69 (46.7%)
		Bolus-correction	73 (49.7%)
		SSI	5 (3.4%)
	Initial TDD, units/kg/day		
		Basal-bolus	0.37 ± 0.14
		Bolus-correction	0.13 ± 0.03
Follow-up			
	Hospital discharge		134 (91.2%)
	Transfer to another ward		20 (13.6%)
	ICU transfer		8 (5.4%)
	Indication of palliative care		6 (4.1%)
	Deaths		13 (8.8%)

Data are mean ± SD or n (%).

BG: blood glucose; BMI: body mass index; eGFR: estimated glomerular filtration rate (CKD-EPI); ICU: intensive care unit; SSI: sliding scale insulin; TDD: total daily dose of insulin.

### Glycemic control

**Figure 1:**
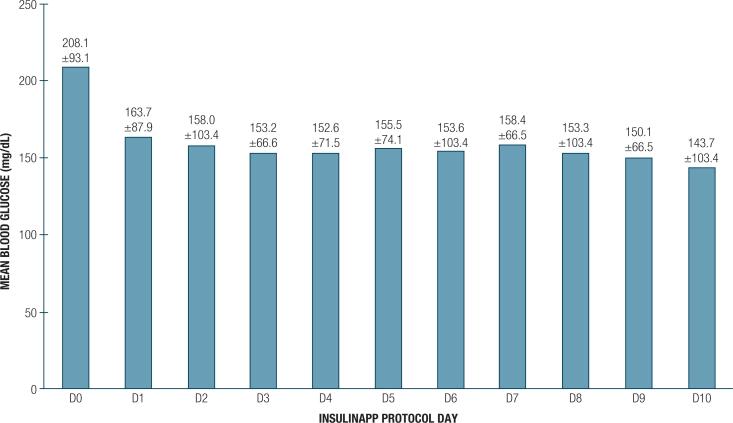
Mean and standard deviation of point-of-care blood glucose throughout hospitalization.

The mean admission BG was 220.3 ± 92.9 mg/dL and HbA1c of 8.3% ± 2.9%. There were 1200 BG-monitored patient-days. Upon hospital admission, 50% patients were treated with bolus-correction regimen, 47% with basal-bolus regimen and 3% with SSI regimen ( Table 1 ). During follow-up, 71% patients required a bolus-basal regimen. Out of 73 patients who started a bolus-correction scheme, 33 (45%) patients had the scheme modified to basal-bolus. Out of five patients who started SSI regimen, 3 switched to bolus-correction regimen and 2 required a switch to basal-bolus regimen. As for the insulin therapy scheme, the median BG at admission was higher in the basal-bolus group and lower in the bolus-correction group. HbA1c medians in these three groups were higher in the basal-bolus group; and BG percentage on therapeutic range was higher in the bolus-correction group ( Table 2 ). There was no statistical difference between these groups regarding sex, age, BMI and cause of admission (Medicine or Surgery). A total of 3316 BG measurements were performed in the first 10 days of the protocol and 2,341 (70.6%) were within the therapeutic range. The evolution of mean daily BG throughout hospitalization is shown on Figure 1 . The BG percentage between 70-180 mg/dL was 70.6%. The mean BG before breakfast, lunch and dinner were 144.1 ± 49.9, 166.1 ± 52.8 and 153.3 ± 46.6 mg/dL, respectively ( *P* < 0.001).

**Table 2:** Comparison of clinical and laboratory characteristics, initial parameters of the InsulinAPP protocol and follow-up of individuals with type 2 diabetes who used basal-bolus, bolus-correction insulin therapy regimens and that required a change from the bolus-correction to basal-bolus regimen in the first ten days of the protocol

Variable		Bolus-correction insulin regimen (n = 33)	Change from bolus-correction to basal-bolus (n = 40)	Basal-bolus insulin regimen (n = 69)	P
Admission BG, mg/dL		148.0 (72.0)	199.5 (86.0)	248.0 (130.5)	<0.001 †
HbA1c, %		6.6% (1.5%)	8.0% (2.8%)	9.4% (4.2%)	<0.001 †
InsulinAPP protocol					
	BG measurements	741	968	1484	
	BG measurements per individual	22.5	24.2	21.5	
	Hypoglycemia episodes	10 (1.3%)	12 (1.2%)	27 (1.8%)	
	% hypoglycemia per individual	0% (0.03%)	0% (0%)	0% (0.04%)	0.586 †
	BG episodes 70-180 mg/dL	647 (87.3%)	730 (75.4%)	871 (58.7%)	
	% BG 70-180 mg/dL	93.3 (14.3)	76.7 (23.5)	65.0 (40.9)	<0.001 †
Follow-up					
	Hospital discharge	28 (84.8%)	38 (95.0%)	63 (91.3%)	0.310 *
	Transfer to another ward	6 (18.2%)	3 (7.5%)	10 (14.5%)	0.428 ‡
	ICU transfer	4 (12.1%)	2 (5.0%)	2 (2.9%)	0.173 ‡
	Indication of palliative care	2 (6.1%)	1 (2.5%)	3 (4.3%)	0.757 ‡
	Deaths	5 (15.2%)	2 (5.0%)	6 (8.7%)	0.310 ‡

Data are median (interquartile range) or n (%).

* Pearson’s chi-squared test.

† Kruskal-Wallis rank-sum test.

‡ Fisher’s exact test.

BG: blood glucose; ICU: intensive care unit.

### Hypoglycemia

A total of 38 (25.9%) patients with diabetes experienced one or more episodes of hypoglycemia (<70 mg/dL), which corresponds to 4.2% patient-days. Among patients with hypoglycemia, 26 (68.4%) had one isolated episode and 12 (31.6%) had two values below 70 mg/dL. The median of percentage BG measurements in patients with hypoglycemia < 70 mg/dL was 5.5% (3.4%). For more severe hypoglycemic events, 7 (4.8%) patients had BG < 54 mg/dL and one (0.7%) had a BG <40 mg/dL. Of the total finger stick BG, 1.5% were from hypoglycemic episodes, 0.8% with BG <54 mg/dL and 0.1% with BG <40 mg/dL. Hypoglycemia was reported in 12 patients (15 BG measurements) on bolus-correction regimen and 26 patients (33 BG measurements) treated with basal-bolus regimen. All seven BG measurements below 54 mg/dL were with the basal-bolus scheme. The statistical analysis comparing the groups with and without hypoglycemia during the first ten days of the protocol is shown in Table 3 . There was no difference between the two groups regarding the clinical and laboratory characteristics, including renal function, and initial parameters of InsulinAPP protocol, except for the highest initial total daily dose of insulin in the basal-bolus regimen in the group that had hypoglycemia ( *P* = 0.02). The indication for palliative care in the group, ICU transfers and in-hospital mortality were not different between the two groups, but the percentage of hospital discharge was higher in the group with hypoglycemia ( Table 3 ). The median percentage of hypoglycemic episodes per individual in each insulin therapy regimen was 0.0% in all groups, with no statistically significant difference between them ( Table 2 ).

**Table 3:** Comparison of clinical and laboratory characteristics, initial parameters of InsulinAPP protocol and follow-up of individuals with type 2 diabetes who had or not hypoglycemia (blood glucose < 70 mg/dL)

Variable			Hypoglycemia (n = 38)	No hypoglycemia (n = 109)	P
Admission BG, mg/dL			194 (172)	204 (97)	0.593 †
HbA1c, %			7.9 (4.6)	8.1 (3.2)	0.975 †
InsulinAPP protocol					
	Initial insulin regimen				0.884 ‡
		Basal-bolus	19 (50%)	50 (45.9%)	
		Bolus-correction	18 (47.4%)	55 (50.5%)	
		SSI	1 (2.6%)	4 (3.7%)	
	Initial TDD, units/kg/day		0.24 (0.20)	0.18 (0.15)	0.316 †
		Basal-bolus	0.35 (0.13)	0.30 (0.05)	0.020 †
		Bolus-correction	0.15 (0.05)	0.15 (0.05)	0.761 †
Follow-up					
	Hospital discharge		36 (94.7%)	98 (89.9%)	0.516 *
	Transfer to another ward		0	20 (18.3%)	0.002 ‡
	ICU transfer		0	8 (7.3%)	0.113 ‡
	Indication of palliative care		0	6 (5.5%)	0.339 ‡
	Deaths		2 (5.3%)	11 (10.1%)	0.516 ‡

Data are median (interquartile range) or n (%).

* Pearson’s chi-squared test.

† Wilcoxon rank-sum test.

‡ Fisher’s exact test.

BG: blood glucose; ICU: intensive care unit; SSI: sliding scale insulin; TDD: total daily dose of insulin.

### Missing data

Out of 3,557 expected BG measurements, 241 (6.8%) were missing. The median percentage of missed BG measurement among patients was 3.9% (0%-10%).

### Outcome of patients after the first 10 days of the protocol

During hospitalization, 91% were discharged home, 14% were transferred to another ward in the hospital complex, 5% required admission to ICU, 4% were indicated for exclusive palliative care and 9% died during their hospital stay ( Table 1 ). Ten (7%) patients died in the hospitalist-managed ward, and five of them were in exclusive palliative care. There was no difference in these outcomes regarding the insulin regimen used in the first ten days of the protocol ( Table 2 ). Of the patients who had at least one episode of BG < 70 mg/dL on the ward, two patients died in the hospital complex and none of them required transfer to ICU ( Table 3 ). Two patients (1.4%) had InsulinAPP protocol interrupted for the individualization of insulin doses throughout the day.

## DISCUSSION

Our retrospective study showed that InsulinAPP application with this use of human insulins is effective and safe in the management of patients with T2D on the hospitalist-managed ward, with more than 70% BG measurements within the therapeutic range and a low rate of hypoglycemia. Computerized physician order entry (CPOE) and clinical decision support (CDSS) systems are important to prevent medication-related errors, to improve quality of care and to increase efficiency in medication administration ( 19 , 21 ). A systematic review analyzed other six applications of insulin therapy in hospitalized patients and these proved to be useful, safe and with improved quality of life for patients, compared to the usual management ( 14 ). Many countries continue to use human insulin formulations (NPH/regular) as a standard of care, thus, the InsulinAPP application represents an important clinical tool for the management of patients with T2D.

We observed that the use of InsulinAPP application resulted in 71% of BG measurements within the glycemic range of 70-180 mg/dL. These results are similar to that of prospective randomized studies evaluating glycemic control and insulin therapy regimens, such as RABBIT2 ( 22 ), DEAN ( 23 ) and Basal Plus ( 5 ) studies.

Standardized insulin order sets are important for improving glucometrics and should be practical and easy to use for greater adherence by the medical staff ( 10 ). We previously reported that InsulinAPP was considered easy to understand and easy to use by physicians from different specialties ( 24 ). The basal-bolus scheme for inpatient insulin therapy is recommended by medical consensus and guidelines ( 1 , 2 ). Some studies have reported on the impact of implementing the basal-bolus regimen as a standard insulin therapy regimen and raised a question about the benefit of using this regimen for all T2D patients ( 25 ). The Society of Hospital Medicine and other studies suggested that a simpler insulin therapy regimen than the basal-bolus might be used initially for T2D individuals with good outpatient glycemic control without insulin therapy or with mild inpatient hyperglycemia ( 26 - 29 ). In agreement with these reports, in individuals with mild hyperglycemia or those who are insulin-naïve or using low doses of outpatient treatment with insulin, the InsulinAPP application recommends an initial approach with bolus-correction regimen ( 16 ). In our study, 47% of the patients did not start insulin therapy with a basal-bolus regimen and around 30% of the total of patients did not need the basal-bolus regimen for glycemic control in the first ten days of hospitalization. Patients with higher HbA1c and BG on admission started a basal-bolus insulin therapy regimen as foreseen in InsulinAPP protocol. The percentage of BG measurements within the range in the first ten days of the protocol was lower in the basal-bolus group, probably due to the higher BG on admission.

Our hospital is a public hospital that is part of the Brazilian national unified public health system ( *Sistema Único de Saúde* – SUS) ( 17 ), in which human insulins (NPH and regular) are the standard treatment defined by the Brazilian Ministry of Health ( 30 ). Pharmacoeconomic studies that evaluated human insulins and insulin analogues have suggested that the use of human insulins are the best options in relation to the cost of treatment ( 31 , 32 ). However, the majority of inpatient insulin therapy studies have been performed with insulin analogues. The DEAN trial ( 23 ) and another randomized study in Paraguay ( 33 ) compared the basal-bolus regimen with insulin analogues and human insulins and reported no difference for improvement in glycemic control. In both studies cited, the human insulins group received two doses of NPH insulin, two-thirds in the morning and one-third before dinner ( 23 , 33 ). In InsulinAPP protocol, NPH insulin is administered equal doses three times a day (before breakfast, before lunch and at bedtime) ( 16 ) and showed similar results to previous randomized controlled studies. Using equal doses of the same insulin can make prescription easier and perhaps also reduce insulin administration errors. NPH and regular insulins can be mixed in the same syringe, making the number of injections similar to insulin regimens with insulin analogues. Our study showed similar hypoglycemia-related metrics as an inpatient glycemic control study from 126 US hospitals ( 34 ).

Around 1% had the protocol interrupted for the individualization of insulin doses throughout the day. As the security mechanism of InsulinAPP application, this recommendation is suggested when there are episodes of hypoglycemia and very high hyperglycemia in the follow-up evaluation ( 16 ).

This study has some limitations, which include those inherent to a retrospective, single-institution study. In this analysis, individuals without diabetes who developed stress hyperglycemia were excluded. In addition, it was not possible to effectively and reliably assess factors that may interfere with HbA1c, such as a history of hemoglobinopathies or recent red blood cell transfusion before admission to our ward. About 75% of the patients included in the study were surgical patients. Therefore, the results should not be generalized to other wards with a different profile of inpatients. The study was not designed to show statistical differences between the group that had hypoglycemia and the one that did not. It was not possible to assess nutritional status, fasting and diet acceptance, which are important predictors of hypoglycemia. The InsulinAPP protocol was not evaluated after the first ten days of the protocol in this study. Thus, the results of the follow-up evaluations do not necessarily represent the conditions of the analysis throughout the entire hospitalization.

In summary, our data indicate that InsulinAPP application is an effective and safe tool for adjusting human insulins doses in hospitalized patients with type 2 diabetes. Not all patients required a basal-bolus insulin therapy regimen and the bolus-correction regimen may be an option for patients with non-severe hyperglycemia. Many countries use human insulins as a standard of care and a protocol using such insulins is of great importance. Furthermore, data such as these are important for the maintenance and continuous improvement of the quality of hospital service.
